# Top-down modulation of gaze capture: Feature similarity, optimal tuning, or tuning to relative features?

**DOI:** 10.1167/jov.20.4.6

**Published:** 2020-04-13

**Authors:** Ashley York, Stefanie I. Becker

**Affiliations:** The University of Queensland, Brisbane, Australia

**Keywords:** visual search, distractor, capture, optimal tuning, relational

## Abstract

It is well-known that we can tune attention to specific features (e.g., colors). Originally, it was believed that attention would always be tuned to the exact feature value of the sought-after target (e.g., orange). However, subsequent studies showed that selection is often geared towards target-dissimilar items, which was variably attributed to (1) tuning attention to the relative target feature that distinguishes the target from other items in the surround (e.g., reddest item; *relational tuning*), (2) tuning attention to a shifted target feature that allows more optimal target selection (e.g., reddish orange; *optimal tuning*), or (3) broad attentional tuning and selection of the most salient item that is still similar to the target (*combined similarity/saliency*). The present study used a color search task and assessed gaze capture by differently coloured distractors to distinguish between the three accounts. The results of the first experiment showed that a very target-dissimilar distractor that matched the relative color of the target but was outside of the area of optimal tuning still captured very strongly. As shown by a control condition and a control experiment, bottom-up saliency modulated capture only weakly, ruling out a combined similarity-saliency account. With this, the results support the relational account that attention is tuned to the relative target feature (e.g., reddest), not an optimal feature value or the target feature.

## Feature similarity, optimal tuning, or tuning to relative features?

The visual world is much richer in information than our severely limited cortical resources can process in real time (Kristjánsson, 2006; Lamy & Kristjánsson, 2013). Visual selection is the mechanism that selects information for further in-depth processing, which allows processing of task-relevant information despite processing limitations (Lamy & Kristjánsson, 2013). Visual selection can be driven either in a bottom-up, stimulus-driven manner (e.g., Theeuwes, 1991; Treisman & Gelade, 1980), or by top-down search strategies that tune attention to goal-relevant attributes (i.e., top-down controlled; Folk & Remington, 1998; Folk, Remington, & Johnston, 1992; Wolfe, 1994).

Bottom-up, or stimulus-driven attentional, capture can occur when a visually salient stimulus attracts attention and the observer's gaze automatically in virtue of its saliency. For instance, previous studies have shown that suddenly appearing objects (abrupt *onsets*) can involuntarily attract attention and the observer's gaze, even when they are completely irrelevant to the task (Hillstrom & Yantis, 1994; Jonides & Yantis, 1988; Theeuwes, 1991, 1994; Yantis & Jonides, 1984; Yantis, 1993; cf. Folk et al., 1992). In addition, it has been reported that items with a high feature contrast can involuntarily capture attention (e.g., Itti & Koch, 2000; Theeuwes, 1992). For instance, in an array of multiple green items, a red item could attract attention and gaze because it is visually salient (i.e., has a high feature contrast) and pops out from the display (e.g., Theeuwes, 1992; but see Becker, 2007).

Importantly, a salient stimulus will attract attention even more strongly when it is similar to the task-relevant target item (Folk et al., 1992). For instance, when searching for a red item, an irrelevant onset will attract attention and the gaze more strongly when it has the same color as the target (red) than when it has another, equally salient color (e.g., Becker, Lewis & Axtens, 2017; Ludwig & Gilchrist, 2002). Stronger capture by target-similar items has been attributed to our ability to bias or *tune* attention to the basic attributes of a sought-after target (e.g., color, size, orientation), which allows limiting attention and gaze movements to potentially task-relevant items (e.g., Becker et al., 2017; Folk & Remington, 1998; Wolfe, 1994).

## Top-down tuning mechanisms

Several different mechanisms have been proposed to explain how exactly attention is top-down tuned to a known target feature. Among the first theories of attention were *feature similarity views* (e.g., *Attentional Engagement Theory,* Duncan & Humphreys, 1989; see also *Feature-Similarity Theory*, Martinez-Trujillo & Treue, 2004), which propose that attention is tuned to the feature value of the target. According to these views, attention would be tuned to orange in search for an orange target, and items should attract attention more strongly the more similar they are to the target [with stronger capture for yellow-orange and red-orange items than for yellow or red items]; e.g., Ansorge & Heumann, 2003; Duncan & Humphreys, 1989; Ludwig & Gilchrist, 2002; Martinez-Trujillo & Treue, 2004). To date, corresponding theories usually predict capture by a combination of top-down tuning to the target feature and the bottom-up saliency of all items (e.g., Itti & Koch, 2000; Ludwig & Gilchrist, 2002; Wolfe, 1994). According to these *combined similarity/saliency views*, items can attract attention and the gaze when they are either visually salient (i.e., when they have a high feature contrast or a unique feature; e.g., Itti & Koch, 2000; Theeuwes, 1992), or when they match the target feature, whereby items that are both salient and similar to the target will attract attention most strongly (e.g., Ludwig & Gilchrist, 2002; Martinez-Trujillo & Treue, 2004).

According to *optimal tuning accounts*, by contrast, attention is not necessarily tuned to the target feature value, but to the feature value that *optimally* distinguishes between the target and the irrelevant nontarget items (e.g., Navalpakkam & Itti, 2007; Scolari & Serences, 2009, 2010). Especially when the target is embedded among similar nontargets, it can be optimal to bias attention to an “exaggerated target feature value” that is slightly shifted away from the nontarget feature values because this reduces the overlap between the feature value distributions and enhances the discriminability of the target (i.e., the signal-to-noise ratio; Navalpakkam & Itti, 2007). For example, if an orange target is embedded among red-orange nontargets, attention would be tuned to yellowish orange, and as a consequence, yellowish orange items should attract attention more strongly than the orange target itself.

The *relational account* (Becker, 2010) could accommodate such a result as well. However, deviating from an optimal tuning account, the relational account claims that attention is usually not tuned to a specific feature value at all, but to *feature relationships* or the *relative feature* of the target, that the target has relative to the other items in the surround (e.g., redder/greener, darker/lighter, larger/smaller). This context-dependent search strategy will typically result in capture by items that have the most extreme feature value that correspond to the target's relative feature (e.g., reddest, darkest, largest item). For instance, in search for an orange target among yellow or yellow-orange items, attention would be tuned to all redder items or the reddest item, and as a consequence, the reddest item in the visual field would capture attention most strongly, followed by the next reddest item, and so on.

Tuning attention to the relative features of items rather than a specific feature value (e.g., larger, darker, redder) is thought to render selection more stable in the natural environment where the exact feature values often change with changes in the distance (e.g., size), perspective (e.g., orientation, shape), and lighting conditions (e.g., shading, clouds).

The relational account and optimal tuning account will often make the same predictions. For instance, when in search for a greenish blue target among green nontargets, both accounts predict stronger capture by a blue distractor than a target-similar bluish green distractor: optimal tuning accounts, because attention should be tuned to a shifted (exaggerated) target feature that will be closer to blue than bluish green (e.g., Navalpakkam & Itti, 2007), and the relational account, because attention is tuned to bluer (or the bluest item), and the blue distractor is bluer than the target and hence, fits the target definition better.

The major difference between the accounts is that optimal tuning only allows for a limited shift, which prescribes that items still need to be visually similar to the target to attract attention or the gaze. By contrast, according to the relational account, capture is predicted to be independent of the similarity to the target, and depends only on whether the item has the same relative feature as the target (Becker, 2010, Becker et al., 2013).

Previous studies testing the relational account against the optimal tuning account used a spatial cueing paradigm, where the distractor is presented before the target in a separate display within a separate cue context. This allows varying the feature similarity and the relative match of cue and target independently of each other (Becker et al., 2013). The results of these studies revealed that a cue that matches the relative color of the target (e.g., bluer) can still capture attention even when it is identical to the nontarget items (e.g., bluish green). This rules out an optimal tuning account and combined similarity/saliency account because attention should be either shifted in the opposite direction, away from the nontarget color (optimal tuning), or centered on the target color (similarity/saliency account). However, these results were obtained in covert attention tasks in which gaze shifts were prevented and attentional capture was inferred by assessing the N2pc in the electroencephalogram of participants (a marker for visual attention; Schönhammer et al., 2017), and behavioral validity effects (i.e., comparing trials in which the singleton cue is presented at a nontarget location vs. the target location; e.g., Folk & Remington, 1998). With this, it is still an open question whether the same effects will be observed in a visual search task when we use eye movements to index attentional capture. As further detailed later, the current evidence in visual search is consistent with optimal tuning, relational tuning, and combined similarity/saliency views.

## The current evidence

**Figure 1.**
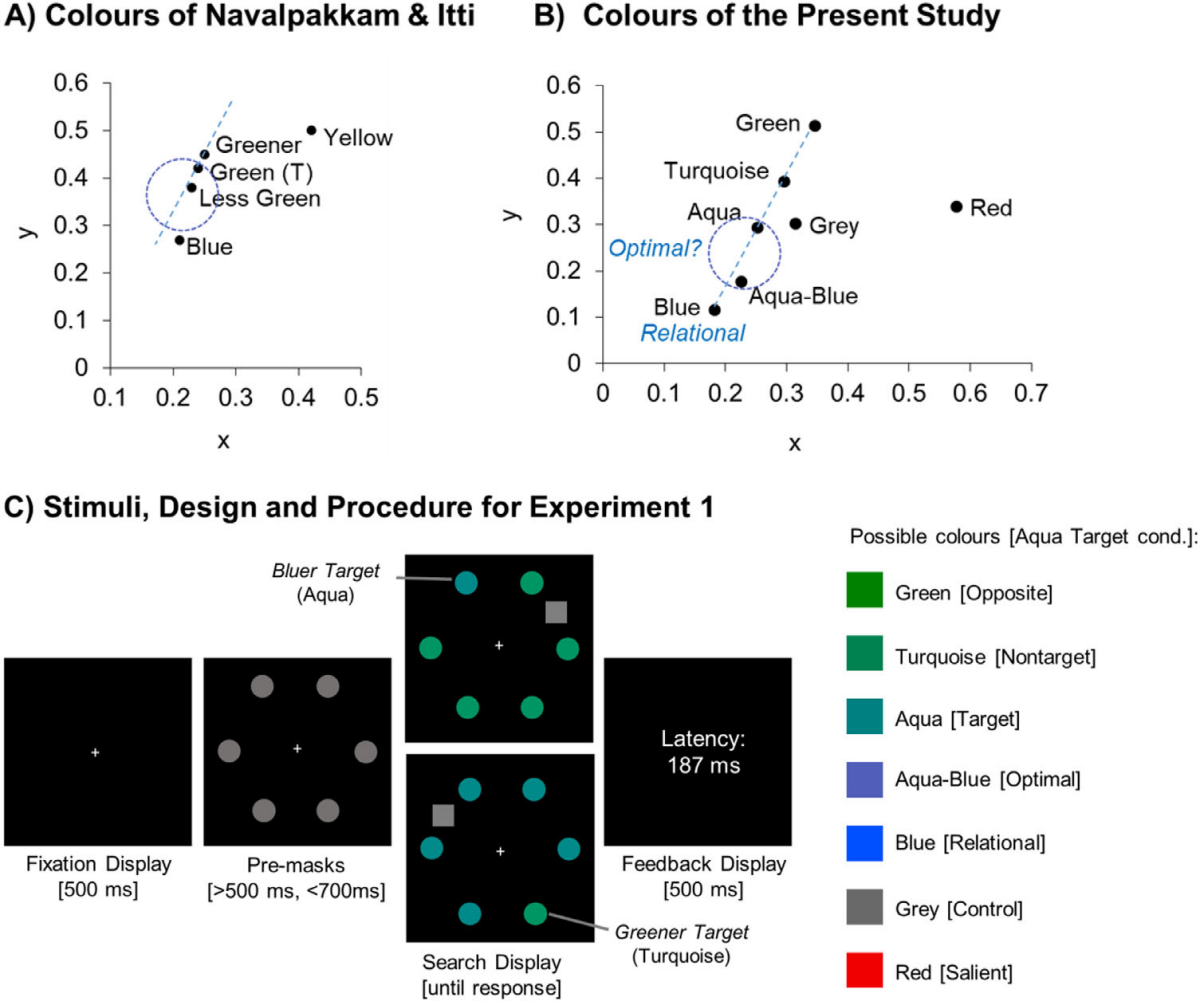
(A) The colors used in the study of Navalpakkam & Itti (2007). (B) The colors used in Experiments 1 and 2, plotted by their coordinates in the CIE 1976 (x, y) color space. The circles depict the possible area for optimal tuning and have the same size in both graphs, and the straight line illustrates the direction in which the target differed from the nontargets (i.e., the relative target feature). (C) Example stimuli and trial sequence for Experiment 1. In different blocks, the target was either aqua (bluer target) or turquoise (greener target), among nontargets of the opposite color. Participants had to make a fast and precise eye movement to the target while ignoring the onset distractor, which was a square that could have one of seven different colors (listed on the right). The distractors were the same in both conditions. Names in brackets refer to the functional color labels in the bluer target condition.

Studies on the optimal tuning account often used a visual search task to bias attention in a particular manner (“training trials”), and then assessed visual performance in probe displays, in which the stimuli were presented only briefly and participants had to pick the target color among four differently colored stimuli. For instance, in Navalpakkam and Itti's (2007) study, observers had to find a medium green target among three greener nontargets that were all quite similar to the target (see Figure 1A for an overview of the colors and their positions in CIE space). Randomly interleaved with these search trials were probe trials, in which participants had to pick out the target color among three other differently coloured probes, two of which were very similar to the target (greenish-blue), and two that were quite dissimilar to the target (yellow, blue; see Figure 1A).

In line with an optimal tuning account, they found that participants most frequently picked an “exaggerated target color” as the target color that was slightly shifted away from the color of the nontargets (“less green” in Figure 1A), 0.03 x/y-units in CIE color space away from the target color. This exaggerated target color was chosen significantly more frequently than the veridical target color (“green”), whereas more target-dissimilar blue and yellow colors that were 0.15 and 0.19 x/y-units away from the target color were chosen not at all or very infrequently (Navalpakkam & Itti, 2007, Figure 1A; see also Scolari & Serences, 2009, 2010). These results were taken to support the optimal tuning account, that attention had been biased toward this slightly shifted feature value, which was more optimal for discriminating and selecting the search target.

The results may also seem inconsistent with a relational account, which would have predicted tuning to all bluer items, and the highest selection rates for the blue color (as it was the bluest item in the display). In line with this contention, previous studies on the relational account found the highest selection rates for distractors that matched the relative color of the target, even when they were quite dissimilar from the target. In one study (Becker et al., 2014; Experiment 3), participants had to search for an orange target among three yellow nontargets (redder target). Attentional biases were assessed by tracking eye movements to differently coloured distractors. The results showed that a red and red-orange distractor both captured the gaze most strongly, and more strongly than a target-similar, orange distractor, despite the red distractor being quite dissimilar from the target color, 0.086 x/y-units away from the target in CIE space. These results were taken to show that attention was tuned to the relative target color (redder), not to the target color, or a slightly shifted or exaggerated target color.

The discrepant findings may be due to differences in the methods, with optimal tuning studies using probe detection and target identification tasks, and relational studies assessing selection by tracking eye movements to distractors. However, a closer look reveals that the results may not be discrepant at all, and are still consistent with either account.

Specifically, the results of the optimal tuning studies may still be consistent with the relational account, because the target-dissimilar blue and yellow probes in Navalpakkam and Itti's (2007) study did not differ in exactly the right direction from the nontargets. This becomes apparent when the colors are plotted by their positions in CIE color space (see Figure 1A). Because blue and yellow both deviate from the direction in which the target differed from the nontargets (indicated by the dotted line in Figure 1A), it is possible that the most extreme colors were not selected because the extreme probes did not have the exact target-matching relative color. Hence, the findings interpreted as support for the optimal tuning account are also consistent with the relational account.

Moreover, the optimal tuning studies also only tested four nontarget probe colors, two of which were similar to the target (0.03 x/y-units away from the target), and two very dissimilar colors (blue, yellow; 0.153 and 0.197 x/y-units away from the target, respectively). This renders it difficult to estimate the magnitude of the proposed shift in tuning. Ultimately, the results allow much larger shifts in tuning than 0.03 x/y units in CIE space, with the upper limit being a 0.0765 x/y-units shift away from the target (less than one-half the distance to the extreme blue probe, to explain why the blue probe was not selected; Navalpakkam & Itti, 2007). With this upper limit, the optimal tuning account could however explain the finding of the relational studies, that target-dissimilar red and red-orange distractors that were 0.068 and 0.086 x/y-units away from the orange target in CIE space showed the strongest capture, and captured the gaze equally strongly (by assuming that the peak of the tuning function is shifted between the two colors). Hence, optimal tuning could explain the findings that were originally interpreted as evidence for the relational account (Becker et al., 2014).

In addition, the results may still be consistent with a combined similarity-saliency account (e.g., Ludwig & Gilchrist, 2002; Martinez-Trujillo & Treue, 2004). Because the irrelevant distractor in the study of Becker et al. (2014) was still quite similar to the target, and yet salient (i.e., different from the target and nontarget items), it is possible that it was selected because it was still coactivated by top-down tuning to the target, and additionally enjoyed a selection advantage because it was more salient than the target-similar distractor. Similarly, a combined similarity-saliency account could also explain higher selection of the moderately shifted probe color in the study of Navalpakkam and Itti (2007). That the blue and yellow probe colors were not selected could then be due to the fact that blue and yellow were too dissimilar to be coactivated by top-down tuning to the target.

## Aim of the present study

The aim of the current study was to critically test which of the three accounts described previously could provide an explanation for the results of previous eye movement studies – the similarity-saliency view, the optimal tuning account, or the relational account. The main limitation of previous studies was that the relatively matching distractors were not sufficiently dissimilar from the target to rule out optimal tuning accounts or combined similarity-saliency views. As described, both accounts may still be consistent with capture by relatively matching distractors in previous studies (e.g., Becker et al., 2014), because (1) the optimal feature may be up to 0.075 x/y-units away from the target, and (2) the corresponding color may still be perceptually similar enough to the target and yet distinct enough so that the distractor profited from being both target-similar and (slightly) salient.

In the present study, we centrally tested gaze capture by a relatively matching (blue) distractor that was perceptually very different from the target color (aqua) and 0.190 x/y-units away from the target color, which was outside the boundaries of optimal tuning (0.153 x/y-units, which marks the location of the blue target-dissimilar probe in Navalpakkam & Itti, 2007). Moreover, we also included a salient red distractor and a nonsalient gray control distractor to assess possible effects of bottom-up saliency on attention.

As in previous studies, capture by the distractor was assessed by monitoring the observer's eye movements during visual search. Specifically, we centrally measured the percentage of first eye movements on the different distractors to index capture by the distractor. As it is possible to shift covert attention without a concomitant eye movement, we also measured the saccade latencies of first eye movements. If there is a shift of covert attention without a concomitant eye movement, this should delay the saccade, as covert attention shifts are time-consuming, and covert attention always needs to shift to a location before an eye movement (Deubel & Schneider, 1996). Hence, if there are distractors that strongly attract covert attention but do not attract the gaze, this should be reflected in elongated saccadic latencies to the target.

## Experiment 1

The aim of the first experiment was to test whether capture by target-dissimilar distractors is best explained by the relational account, the optimal tuning account, or a combined similarity-saliency view. In the experiment, observers had to search for a particular, predefined color target, and we assessed capture by differently colored onset distractors by monitoring the observer's eye movements during search (for a similar approach see Becker & Lewis, 2015; Becker, Lewis, et al., 2017; Born, Kerzel, & Theeuwes, 2011; Martin & Becker, 2018; Mulckhuyse, Van Zoest, & Theeuwes, 2008).

Experiment 1 comprised two blocked conditions: an experimental, *bluer target* condition, designed to distinguish between the three accounts, and a *greener target* control condition.

In the bluer target condition, the target had an aqua (bluish green) color, and was presented among turquoise (greenish) nontargets. Participants had to make a fast and accurate eye movement to the target and ignore an onset distractor that could have one of seven different colors (see Figures 1B, 1C). The distractor also always had a different shape as the other search items (to avoid confusing it with the target; see Becker et al., 2014, for a similar procedure). The colors were all equiluminant and are shown by their respective positions in CIE color space in Figure 1B. Critically, the distractor colors were chosen such that the three accounts would arrive at different predictions about which one of them would attract the gaze most strongly: a target-similar (aqua) distractor, intermediate aqua-blue distractor, or blue distractor.

The *blue distractor* matched the relative color of the target (bluer), but was quite dissimilar from the target, differing by 0.19 units in x/y CIE color space from the target. With this, the blue distractor was further away from the target as the blue probe in the optimal tuning study (0.153 x/y-units in CIE color space; see Figures 1A, 1B and Navalpakkam & Itti, 2007), and outside the theoretically maximum possible shift in optimal tuning (0.075 x/y units in CIE space). However, it differed in the correct direction from the target and nontargets (placed on the line distinguishing the target from the nontarget color). Hence, according to the relational account, the blue distractor should capture the gaze most strongly. A corresponding finding would provide strong evidence for the relational account and against the optimal tuning account.

**Figure 2.**
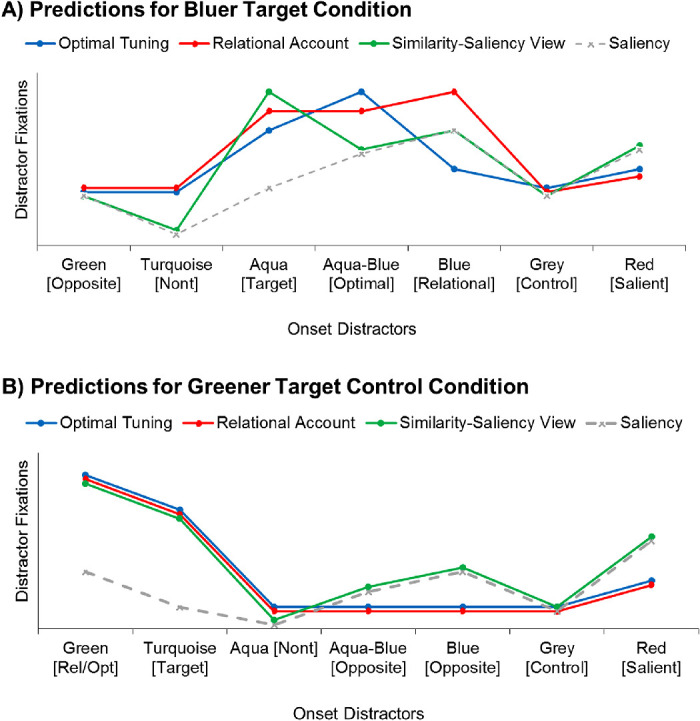
(A) Predicted selection rates of all distractors in the greener target condition (turquoise target), depicted separately for an optimal tuning account, relational account, and a combined similarity-saliency view. (B) Predicted selection rates of the bluer target condition (aqua target), when attention is tuned to an optimal color, relative target color, or the exact color of the target (similarity-saliency view).

The *intermediate aqua-blue distractor* was placed 0.119 units in x/y CIE color space away from the target and was shifted slightly off the direct line in which the target differed from the nontargets (similar to the optimally placed, “less green” probe in the study of Navalpakkam & Itti, 2007). Thus, if attention is tuned to an optimal color that is shifted as much as 0.06 x/y-units away from the target, this distractor should attract attention most strongly – more strongly than the target-similar aqua distractor and the target-dissimilar blue distractor. A corresponding finding would provide strong evidence for an optimal tuning account. Figure 2A depicts the predictions of the two accounts (relational, optimal) across all distractor conditions.

The predictions of a combined similarity/saliency account are more difficult to derive because they depend on the relative contributions of top-down tuning versus saliency, and on the width of the top-down tuning function. In the bluer target condition, it is perhaps unlikely that attention would be tuned so broadly to the target color as to coactivate the colors of the intermediate aqua-blue or blue distractor because they were quite dissimilar from the target color (e.g., more dissimilar than in the study of Navalpakkam & Itti, 2007), and more dissimilar than the nontargets). Therefore, the combined similarity/saliency account would probably predict top-down activation only for the target color (aqua). If capture is predominantly determined by top-down activation, the target-similar (aqua) distractor should capture most strongly.

However, if capture is more strongly determined by bottom-up saliency, the distractors with the highest feature contrasts should attract the gaze most strongly (e.g., Becker, Lewis & Axtens, 2017; Folk & Remington, 1998). To gauge possible contributions of bottom-up saliency to capture, we included a very salient red distractor. The red distractor had a similar feature contrast as the relatively matching, blue distractor (0.299 and 0.288 x/y-units in CIE space away from the nontarget items, respectively). To test whether capture is modulated by bottom-up saliency, we centrally compared the capture effects of the salient red distractor with a nonsalient gray distractor (see Figure 1B). Moreover, in addition to the predictions of a combined similarity/saliency account, we also depicted the bottom-up saliency of all distractors in Figure 2A (computed as the mean distance of each distractor color to the search items; i.e., the five nontargets and the target color).

As shown in Figure 2A, the three accounts should all make different predictions about the peak of the capture effects, with the relational account predicting the strongest capture effect for the dissimilar, blue distractor, the optimal tuning account predicting the strongest capture for the intermediate aqua-blue distractor, and the similarity-saliency account predicting the strongest capture for the target-similar aqua distractor. Naturally, these predictions depend on a number of assumptions about each of the accounts and about CIE color space accurately reflecting perceptual similarity relations. These assumptions will be tested in a control experiment (Experiment 2). In addition, Experiment 1 included a greener target control condition to test whether the effects generalize to different colors.

In the greener target condition, we reversed the target and nontarget colors so that observers now searched for a turquoise (bluish green) target among aqua (greenish blue) nontargets. The distractors had the same colors as in the bluer target condition. The most important distractor in this condition was the green distractor, which was greener than the target and thus, relatively matched the target (i.e., differed in the right direction from the target), and was 0.131 x/y-units away from the target color in CIE space.

According to the relational account (Becker, 2010), the green distractor should attract attention and the gaze more strongly than the target-similar turquoise distractor because attention is tuned to the greenest item in search for the target, and the green distractor, if present, is the greenest item. However, a corresponding result would also still be consistent with an optimal tuning account, if attention can be shifted as much as 0.075 x/y units in CIE space (as this would result in stronger capture by a green than turquoise distractor). Moreover, a combined similarity-saliency view could also still account for these results, as green may still be similar enough to the target color (turquoise) to be included in a broad top-down setting, and is simultaneously more salient than the target color (turquoise; see Figure 1B). The predicted capture effects of the optimal tuning account, relational account, and combined similarity/saliency view are depicted in Figure 2B, along with the bottom-up saliency of all distractors (computed as the mean feature contrast of each distractor color to the search items [i.e., the color of the five nontargets and the target]).

To assess capture by each of the distractors, we centrally measured the percentage of first eye movements that directly went to each distractor. Moreover, to assess whether any of the distractors attracted covert attention without a concomitant gaze shift, we assessed the target saccade latencies – that is, the time from the onset of the target display to the first saccade that directly went to the target. Because covert attention shifts are time-consuming, covert attention shifts to a distractor should be reflected in longer target saccade latencies (e.g., Becker, 2010; Becker et al., 2017; Deubel & Schneider, 1996).

### Method

#### Participants

Twenty-three participants (three male, 20 female) with a mean age of 23.09 years (*SD* = 4.84 years) and normal or corrected-to-normal vision participated in the experiment. The participants were naïve to the purpose of the experiment and compensated with $10AU/h for their time. The procedures of the study were approved by the Ethics Committee of The University of Queensland, Australia, and were line with the ethical principles of experiments involving humans outlined in the Declaration of Helsinki.

#### Apparatus

A Dell OptiPlex 745 computer (Dell, Texas) and a BenQ 19-in. LCD color monitor (BENQ, Taipeh) were used for the experiment. All stimuli were presented on a monitor with a resolution of 1280 × 1024 pixels and a refresh rate of 75 Hz. A video-based eye tracker (Eyelink 1000, SR Research, Ontario, Canada) recorded eye movements with a spatial resolution of 0.1° and a temporal resolution of 500 Hz. A standard mouse was used to record responses while observers viewed the screen from a distance of 62 cm. Viewing position was fixed using a headrest and the chinrest of the eye tracker. Presentation software (Neurobehavioral Systems) controlled the sequence of trials in the experiment and provided performance feedback during the experiment.

#### Stimuli

All stimuli were presented against a black background. The fixation display consisted of a white cross (size: 0.27° × 0.27°) presented at the center of the screen. The premask display consisted of the fixation cross and 6 colored disks (diameter: 1.38°) that were distributed evenly on the outlines of an imaginary circle with a diameter of 17.7° (see Figure 1C). In the search display, one of the gray premasks changed to the target color (aqua or turquoise), whereas the remaining disks changed to the nontarget color (turquoise or aqua, respectively). The onset distractor was a colored square that appeared in a previously empty location between two nontarget items. The colors were matched for luminance with a CRS ColourCal MKII colorimeter and had the following luminance and CIE (1976) color values (L*x,y*): blue [RGB: 0, 80, 195], 17.08, .180, .117; aqua-blue [RGB: 82, 95, 186]: 16.26, .226, 0.177; aqua [RGB: 0, 128, 128]: 17.88, .251, .294; turquoise [RGB: 0, 130, 80]: 17.01, .295, .395; green [RGB: 0, 130, 0]: 16.01, .346, .515; gray [RGB: 105, 105, 105]: 15.42, .314, .303; and red [RGB: 240, 0, 0]: 16.41, .577, .340.

#### Design

Experiment 1 had a 2 (target color) × 7 (distractor color) within-subjects design. The color of the targets and nontargets was blocked, whereas the color of the onset distractor varied randomly within each block, with the restriction that all distractor colors were presented an equal number of times. The target and distractor positions were chosen randomly on each trial, with the provision that the distractor was never positioned directly adjacent to the target (see Mulckhuyse et al., 2008, for a similar design). Participants completed 560 trials in total, 80 with each distractor color.

#### Procedure

The experiment was conducted in a normally lit room. Participants were instructed to make a fast and precise eye-movement to the target (defined by a color change from gray to aqua or turquoise), and to press a mouse-button while they were still fixating on the target. Participants were informed of the possible distractors in advance, and told to ignore them as much as possible.

Each trial started with the presentation of the fixation display (500 ms), followed by the premask display. The target display was only presented when participants had maintained fixation on the fixation cross (within an area of 1.36°), for at least 500 ms, plus a random period between 1 and 200 ms. The search display was presented until the observer's manual response. Immediately after the response, participants received written feedback detailing their saccade latency (i.e., the time from the onset of the target to the point in time that the first eye movement started). If the gaze was still within the fixation area after 300 ms had elapsed (from the onset of the search display), the words “Too Slow” were additionally displayed below the saccade latency feedback. The feedback was implemented to discourage participants from searching covertly for the target and delaying their eye movements. Participants were given regular breaks throughout the experiment.

### Results

#### Data

Eye movements were parsed into saccades, fixations, and blinks, using the standard parser configuration of the Eyelink software, which classifies an eye movement as a saccade when it exceeds a velocity of 30°/s or an acceleration of 8,000°/s. The first eye movement on a trial was attributed to the nearest stimulus (target, distractor, or nontarget) when it was outside the fixation area of 200 pixels around the fixation cross. Saccade latencies were computed from the onset of the trial to the point in time when the saccade started, according to the velocity and acceleration criteria. Trials with first saccade latencies below 100 ms or above 1,000 ms of stimulus onset were excluded (0.58%), as were trials in which the first eye movement could not be assigned to a stimulus (11.4%). Three subjects were excluded because their data loss exceeded 25%.

#### Percentage of first saccades to the distractor

**Figure 3.**
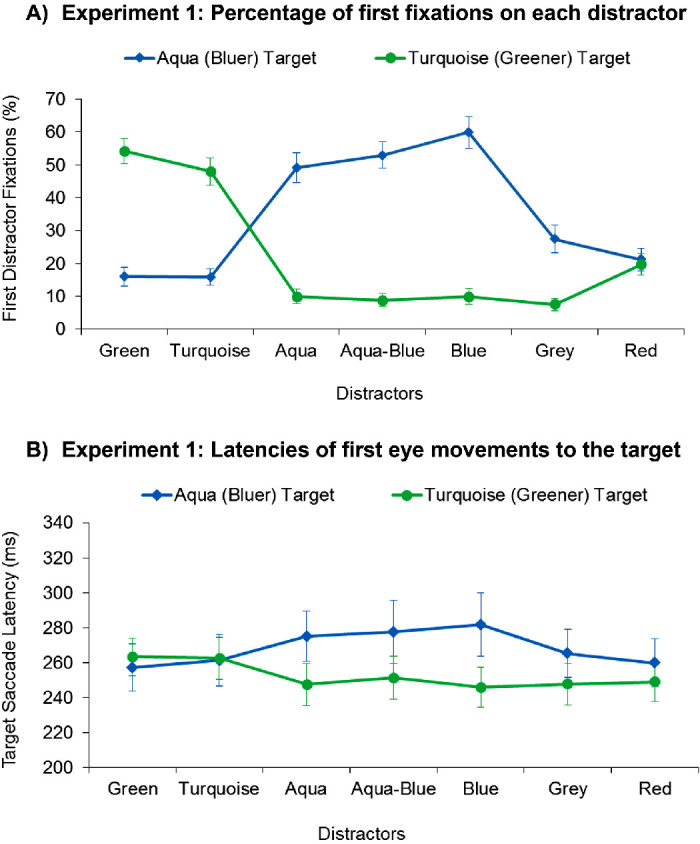
(A) The percentage of first eye movements to each of the different distractors observed in Experiment 1, depicted separately for the different distractors, and the aqua target and turquoise target conditions. (B) The mean latencies for the first saccades in a trial directly went to the target showed the same results as the percentage of first fixations to the distractors, indicating that the results were not due to a speed-accuracy trade-off. Error bars depict ± 1 *SEM*.

The majority of first eye movements were directed to the target or the distractor (>89% of first saccades across all conditions). A 2 × 7 repeated-measures analysis of variance (ANOVA) with the variables “target color” (bluer/aqua, greener/turquoise) and “distractor color” (blue, aqua-blue, aqua, turquoise, green, red, gray) showed a significant main effect of target color, *F*(1, 19) = 29.35, *p* < 0.001, *ƞ^2^_p_* = .607, such that overall, observers made more first eye movements to distractors in the bluer/aqua target condition (*M* = 34.67%) than in the greener/turquoise target condition (*M* = 22.63%). In addition, we found a main effect of distractor type, *F*(6, 114) = 23.79, *p* < 0.001, *ƞ^2^_p_* = .556, and a significant interaction between target color and distractor type, *F*(6, 114) = 101.75, *p* < 0.001, *ƞ^2^_p_* = .843 (see Figure 3A), reflecting that the distractors captured the gaze differently, depending on the target condition.

The results of the critical, bluer target condition are displayed in Figure 3A. As shown in the graph, the relatively matching (blue), intermediate (aqua-blue), and target-similar (aqua) distractors all attracted the gaze most strongly, more strongly than the other four distractors, all *t*s > 6.57, *p*s < 0.001. To test which of the three distractors captured most, we compared the distractor fixations across the three distractors with paired, two-tailed *t*-tests. As predicted by the relational account, the relatively matching (blue) distractor attracted the gaze most strongly, significantly more strongly than the intermediate (“optimal”), aqua-blue distractor, *t*(19) = 2.29, *p* = 0.034, and the target-similar (aqua) distractor, *t*(19) = 2.92, *p* = 0.009. By contrast, the intermediate (optimally placed), aqua-blue distractor and the target-similar (aqua) distractor did not differ, *t*(19) = 1.03, *p* = 0.317. These results demonstrate that a relatively matching, blue distractor can attract attention most strongly, even when it is very dissimilar from the target, and outside the area of optimal tuning, which supports the relational account over an optimal tuning account or combined similarity-saliency view.

To gauge possible contributions of bottom-up feature contrast (saliency) to capture, we also compared the capture rates of the remaining 4 distractors. Of these, the nonsalient gray control distractor was selected most frequently, significantly more frequently than the salient red distractor, *t*(19) = 2.35, *p* = 0.030, the nontarget-similar (turquoise) distractor, *t*(19) = 4.18, *p* = 0.001, and the green distractor, *t*(19) = 5.16, *p* < 0.001. The salient red distractor was also selected more frequently than the nontarget-similar (turquoise) distractor, *t*(19) = 3.47, *p* = 0.003, and the green distractor, *t*(19) = 2.59, *p* < 0.001, whereas the latter two distractors did not differ from each other, *t* < 1. The finding that the nonsalient gray distractor attracted the gaze more strongly than the salient red distractor indicates that bottom-up saliency did not (strongly) drive visual selection, indicating that the high capture rates of the target-dissimilar blue distractor cannot be attributed to its higher bottom-up saliency.

The results of the greener (turquoise) target condition are depicted in Figure 3A, and showed the strongest capture effects for the target-similar (turquoise) and green distractor, which was predicted to strongly attract the gaze by all three accounts, all *t*s > 7.66, *p*s < 0.001 (compared with the other five distractors). In line with the prediction of all three accounts, the green distractor attracted the gaze more strongly than the target-similar (turquoise) distractor, *t*(19) = 2.12, *p* = 0.047.

Of the remaining five distractors, the red distractor attracted gaze most strongly, significantly more strongly than the aqua-blue, gray, aqua, or blue distractors, all *t*s > 3.23, *p*s ≤ 0 .004, whereas the latter four distractors did not differ significantly from each other, all *t*s < 1.57, *p*s > 0.13. Because the red distractor was the most salient item, this finding is consistent with a bottom-up saliency effect. However, the blue distractor was also quite salient, yet did not attract the gaze more strongly than the nonsalient distractors, indicating that bottom-up saliency did not (strongly) drive visual selection.

#### Saccadic latencies to target

To examine whether covert attention may have been shifted to one of the distractors without shifting the gaze, we also analyzed the mean saccade latencies of first eye movements to the target.^1^ The same 2 (target color) × 7 (distractor color) ANOVA computed over the mean saccade latencies showed a main effect of target color, reflecting that the greener (turquoise) target was selected earlier (*M* = 252 ms) than the bluer (aqua) target (*M* = 268 ms), *F*(1, 19) = 7.10, *p* = 0.015, *ƞ^2^_p_* = .27. There was no main effect of distractor color, *F*(6, 114) = 1.87, *p* *=* 0.13, but a significant interaction between the variables, *F*(6, 114) = 10.91, *p* *<* 0.001, *ƞ^2^_p_* = .365, showing that the distractors affected target selection differently, depending on the target condition.

In search for the bluer (aqua) target (blue line graph of Figure 3B), the latencies closely matched the gaze capture results: Target fixation latencies were longest in the presence of the relatively matching (blue) distractor, the optimal (aqua-blue), and the target-similar (aqua) distractor, significantly longer than in the presence of the other four distractors, all *t*s > 2.1, *p*s ≤ 0.048 (with the exception of the intermediate aqua-blue distractor, which did not differ from the gray distractor, *t*(19) = 1.71, *p* = 0.103). Target fixation latencies also did not differ significantly between the relatively matching (blue), intermediate (aqua-blue) and target-similar (aqua) distractors, *t*s < 1.

Similarly, the target fixation latencies did not differ between the remaining four distractors (nonsalient gray distractor, salient red distractor, nontarget-similar turquoise distractor, and green distractor), all *t*s < 2.1, *p*s > 0.05. These results broadly mimic the findings from the distractor fixations, and indicate that those distractors that captured the gaze most strongly also attracted covert attention most strongly, which delayed the first eye movement to the target.

The latencies for selecting the turquoise target are depicted in Figure 3B, and were similar to the gaze capture results: The longest latencies were recorded in the presence of the relationally best (green) and target-similar (turquoise) distractors, whereby the latencies did not differ between these conditions, *t* < 1. In both conditions (green, turquoise), target saccade latencies were significantly longer than in any of the other distractor conditions (aqua-blue, aqua, blue, gray, red), all *t*s > 2.75, *p*s ≤ 0.31, and these conditions did not differ significantly from each other (aqua-blue, aqua, blue, gray, red), all *t*s < 1.7, *p*s > 0.10.

Taken together, the target saccade latencies showed a similar results pattern to the percentage of distractor fixations, reflecting that those distractors that attracted gaze most strongly also significantly delayed eye movements to the target. The delay presumably reflects that covert attention was shifted to the distractor, which could be quickly rejected, preventing a gaze shift to the distractor and allowing selecting the target with the first eye movement. With this, interpretation of the results is not complicated by speed-accuracy tradeoffs or covert attention behaving differently from overt eye movements.

### Discussion

The results of Experiment 1 provided evidence for a relational account, in which attention is tuned to the relative feature of the target and capture is independent of target similarity (Becker, 2010; Becker et al., 2013; Becker, Harris, et al., 2017). The blue distractor was very dissimilar from the target, and differed by more than twice the distance in feature space from the target in previous comparable studies (e.g., Becker et al., 2014); yet captured attention and the gaze more strongly than more target-similar distractors. These results are difficult to reconcile with the claim that attention is tuned to the target feature value (e.g., Duncan & Humphreys, 1989; Martinez-Trujillo & Treue, 2004), or a feature value that is slightly shifted away from the target (i.e., optimal tuning; Navalpakkam & Itti, 2007; Scolari & Serences, 2009).

For an optimal tuning account to explain stronger capture by blue than aqua-blue and aqua, it would have to assume a shift that covers more than 0.155 x/y-units in CIE space because only a shift of that magnitude (or larger) would lead to stronger capture by the blue distractor than the intermediate aqua-blue distractor (because the peak of the tuning function needs to be closer to blue to produce stronger capture by blue [i.e., 0.119 x/y-units + (0.190 – 0.119 x/y units)/2]).

Yet, the results of Navalpakkam and Itti (2007) showed that a target-dissimilar blue probe in their study that was 0.153 x/y-units away in CIE space was not selected. Hence, assuming such a large shift would render the optimal tuning account inconsistent with the results that originally motivated the optimal tuning account (Navalpakkam & Itti, 2007).

The finding of stronger capture by blue than aqua-blue and aqua is also difficult to reconcile with a combined similarity-saliency account, which centrally assumes that attention is broadly tuned to the target color (aqua), with coactivation of target-similar colors. Aqua-blue and blue were chosen to be quite dissimilar to the target color (aqua; see Figure 1B), and were also quite far away from the target color in CIE color space. If a similarity-saliency account would propose coactivation of blue by tuning to the target color, this would result in a very broad attentional tuning function spanning 0.19 x/y-units in CIE space in all directions (given that tuning functions are usually assumed to be symmetrical; e.g., Scolari & Serences, 2009). This would result in tuning attention to a vast array of different colors, ranging almost from full blue to full green, which would render it difficult to discriminate the aqua target from the turquoise nontargets

Stronger capture by the target-dissimilar blue than target-similar aqua distractor is also unlikely to be due to the larger bottom-up saliency of the blue distractor. As shown in the present results, the data did not show a correspondingly large saliency effect. Whereas the red salient distractor attracted the gaze more strongly than several nonsalient distractors, it only attracted 5.4% more first fixations than the least salient distractor (i.e., the nontarget-colored turquoise distractor). Moreover, in the bluer target condition, the nonsalient gray distractor captured the gaze more strongly than the salient red distractor. With this, the saliency effect seems too small and not reliable enough to explain the large capture effect of the blue distractor. Rather, stronger capture by the (not very salient) target-similar distractor than the salient red distractor (mean difference: 27.9%) argues for an account where top-down tuning is the more important determiner of capture (e.g., Folk & Remington, 1998) than bottom-up saliency (e.g., Theeuwes, 1992).

That said, the moderate bottom-up saliency effect could potentially explain stronger capture by blue than the target-similar distractor if we assume equal top-down tuning to aqua and blue. This raises the question whether our assumptions about the similarity between the different colors were correct. Our reasoning centrally relies on distances in CIE color space, which is generally assumed to represent the degree of perceptual similarity between colors. However, if, contrary to this, the relatively matching, blue distractor was more similar to the aqua target than the intermediate, aqua-blue distractor, a combined similarity-saliency account or the optimal tuning account may still be able to account for the results. This is all the more so because we mixed a small amount of red into the color of the aqua-blue distractor (to shift if off the direction in which the target differed from the nontargets and reduce its attention-driving capacity).

Experiment 2 was designed to address this possibility, by encouraging observers to tune attention to the exact target color (i.e., engage in feature-specific search), which should allow assessing the similarity relations between the colors experimentally.

## Experiment 2

Experiment 2 used the same task and stimuli as the bluer (aqua) target condition of Experiment 1. However, observers were encouraged to tune attention to the specific feature value of the target. This was achieved by blocking the distractor conditions, so that the distractor color always remained constant and was consistently repeated within a block of trials. Consistently presenting a blue or an aqua-blue distractor will prevent a relational search strategy in the relevant conditions (with a blue or intermediate aqua-blue distractor), because the target is no longer the bluest item in the display (but only the second-bluest item). Similarly, consistently presenting a blue or aqua-blue distractor will also discourage shifting attention to a different color, as shifting attention away from the nontarget color would result in tuning attention to the blue or aqua-blue distractor.

Thus, observers should adopt a feature-specific search strategy in the critical distractor conditions, where attention is as closely tuned to the specific target color as possible (Becker et al., 2014). Corresponding results have also been found in previous studies that rendered a relational search strategy impossible: When an orange target was not reliably the reddest item in the visual field anymore, rather than observing the strongest capture by red and red-orange, a target-similar orange distractor attracted the gaze most strongly (Becker et al., 2014; see also Harris et al., 2013). However, attention was rather broadly tuned to the target color, with high gaze capture rates for target-similar colors, especially those that were shifted away from the nontarget color, resulting in an asymmetric results pattern (favoring red-orange over yellow-orange; Becker et al., 2014; see also Navalpakkam & Itti, 2007; Scolari & Serences, 2009).

In the present study, we would expect a similar result for the blocked distractor conditions, with more capture for target-similar colors, especially perhaps those that are slightly shifted away from the nontargets. Thus, if our original assumption holds, that aqua-blue is more similar to the target color than blue, the results should now show stronger capture by the aqua-blue distractor than the blue distractor. If, on the other hand, the metrics of CIE feature space do *not* reflect the underlying perceptual similarity relations (despite the fact that the colors are arranged in CIE space to reflect similarity relations between colors), and the blue distractor is more similar to the target, we should observe the same results as in Experiment 1, with stronger capture for the blue distractor than the aqua-blue distractor.

### Method

#### Participants

Twenty-three participants from the University of Queensland, Australia, participated in the experiment. The 23 participants (10 male, 13 female; 2 left-handed, 21 right-handed) had a mean age of 21.70 years (*SD* = 1.82 years) and had all normal or corrected-to-normal vision. Participation was voluntary and participants were compensated with $10AU/h for their time.

#### Apparatus and stimuli

These were the same as in Experiment 1.

#### Design

Experiment 2 ^6^ contained only a the bluer target condition in which an aqua target was presented among homogeneous turquoise nontargets. The distractor color was also blocked, with the order of blocks being randomly determined. The distractor identities were the same as in Experiment 1, resulting in a 1 × 7 within-subjects design (560 total trials, 80 per distractor color).

#### Procedure

The experiment followed the same procedure as Experiment 1, with the addition that participants were informed explicitly about the distractor color before each distractor block and instructed to use this knowledge to ignore the distractors.

### Results

#### Data

Data were processed as in Experiment 1. One of the 23 participants was removed because of not completing the experiment. Excluding trials in which the first eye movement had a latency shorter than 100 ms or longer than 1,000 ms led to a loss of 0.85% of all data, and excluding trials in which the first eye movement could not be assigned to a stimulus (outside the fixation area) led to the exclusion of 8.32% of all data.

#### Percentage of first saccades to the distractor (blocked)

As in Experiment 1, the majority of first eye movements was directed either to the target or the distractor (>92% of first saccades across all conditions), indicating that the target could be located efficiently.

**Figure 4.**
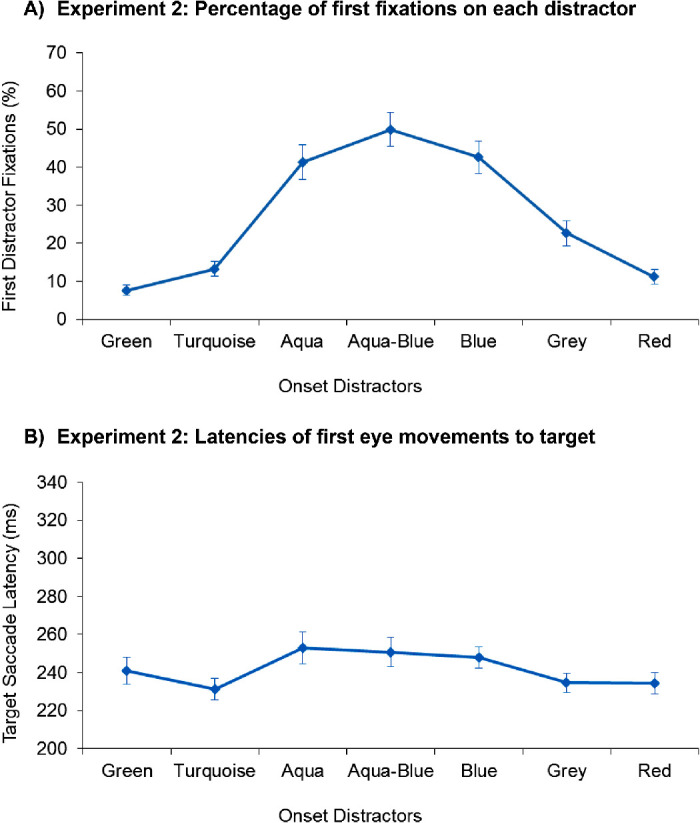
(A) The percentage of first eye movements to each of the different distractors observed in Experiment 2, depicted separately for the different distractors. (B) The mean target saccade latencies, which is the time needed to initiate a saccade to the target when the target was the first item selected. The error bars indicate ± 1 *SEM*.

The results of Experiment 2 are depicted in Figure 4A. Analyzing the percentage of first distractor fixations with a one-way ANOVA showed a significant main effect of distractor type, *F*(6, 126) = 44.58, *p* < 0.001, *ƞ^2^_p_* = .680. Pairwise *t*-tests revealed that the aqua-blue distractor captured the gaze most strongly, significantly more strongly than the target-similar aqua distractor, *t*(21) = 2.74, *p* = 0.012, and marginally significantly more strongly than the blue distractor, *t*(21) = 1.85, *p* = 0.079. By contrast, capture by the target-similar distractor did not differ from the blue distractor, *t* < 1 (see Figure 4A).

As in Experiment 1, the effective distractors (blue, aqua-blue, and aqua) were all selected significantly more frequently than the remaining four distractors; i.e., the nontarget-similar (turquoise), green, gray, or red distractors; all *t*s > 5.48, *p*s < 0.001. Among the latter distractors, the gray distractor was selected more frequently than the other three distractors (red, green, turquoise), all *t*s > 2.63, *p*s ≤ 0.016. In addition, the nontarget-similar (turquoise) distractor was selected more frequently than the green distractor, *t*(21) = 2.91, *p* = 0.008 (all other *p*s > 0.07). These results validate our original assumption, that the intermediate (aqua-blue) distractor was more similar to the target color (aqua) than the more extreme blue distractor.

#### Saccadic latencies to target

To test for covert attention shifts to the distractors that were not accompanied by a gaze shift, we computed the same one-way ANOVA over the mean saccadic latencies. The results showed a main effect of distractor color, *F*(6, 126) = 3.35, *p* = 0.016, *ƞ^2^_p_* = .138 (Greenhouse-Geisser corrected). As shown in Figure 4B, the target saccade latencies were longest in the presence of a target-similar distractor, followed by the aqua-blue distractor and the blue distractor, whereby none of the differences was significant, all *t*s < 1. Target saccade latencies were significantly longer in the presence of any of these distractors (aqua, aqua-blue, blue) than in the presence of the nontarget-similar turquoise, gray, and salient red distractor, all *t*s > 2.21, *p*s ≤ 0.038, though not the green distractor, which did not differ from any distractors, all *t*s < 1.94, *p*s > 0.066. Target saccade latencies also did not differ between the turquoise, green, gray, or red distractors, all *t*s < 1.94, *p*s > 0.066.

Collectively, these results indicate that the distractors that attracted gaze most strongly also delayed target selection most strongly, thus providing no indication for a speed-accuracy tradeoff, or differences in covert attention shifts and eye movements to the distractors.

### Comparison Experiments 1 and 2

To check whether Experiments 1 and 2 indeed yielded different results indicative of different search modes, we also compared the distractor effects across experiments. A 2 × 7 ANOVA with the between-subjects factor of “experiment” (Exp. 1 vs. 2) and the within-subjects factor of distractor color (blue, aqua-blue, aqua, turquoise, red, gray, green) computed over the distractor selection rates of the bluer target conditions showed a significant main effect of distractor color, *F*(6, 240) = 97.78, *p* < 0.001, *ƞ^2^_p_* = .710, and of experiment, *F*(1, 40) = 4.10, *p* < 0.050, *ƞ^2^_p_* = .093, but no significant interaction, *F*(6, 126) = 1.96, *p* = 0.114, *ƞ^2^_p_* = .047 (Greenhouse-Geisser corrected). As no significant differences were expected among the noneffective distractors, we repeated the analysis including only the critical distractors (blue, aqua-blue, and aqua). The results still showed a significant main effect of distractor color, *F*(2, 80) = 3.85, *p* < 0.025, *ƞ^2^_p_* = .088, no difference between experiments, *F*(1, 40) = 2.85, *p* = 0.099, and now also a significant interaction, *F*(2, 80) = 4.06, *p* = 0.021, *ƞ^2^_p_* = .092. Comparing distractor selection rates across the experiments with independent pairwise *t*-tests revealed that the blue distractor captured significantly more strongly in Experiment 1 than in Experiment 2, *t*(40) = 2.67, *p* = 0.011, whereas capture by the aqua-blue and target-similar distractors did not differ, both *t*s < 1.3, *p*s > 0.230.

The same 2 × 7 ANOVA computed over the target saccade latencies revealed only a significant main effect of distractor color, *F*(6, 240) = 7.5, *p* < 0.001, *ƞ^2^_p_* = .158, but no effect of Experiment, *F*(1, 40) = 3.13, *p* = 0.084, and no significant interaction, *F* < 1. The results were the same when only the three critical distractors were included in the analysis (blue, aqua-blue, aqua), indicating no signs of a speed-accuracy tradeoff.

### Discussion

In Experiment 2, we used the same colors as in Experiment 1 and blocked the distractors, which prevents a relational search strategy in the critical conditions (blue and aqua-blue distractor) and encourages tuning attention to the specific target color (e.g., Becker et al., 2014; Harris et al., 2013). In line with this contention, the results showed more capture by the intermediate, aqua-blue distractor than by the blue or aqua distractor. This indicates that attention was not tuned to all bluer items or the bluest item, but to a limited area in feature space, which was closest to aqua-blue, rendering it difficult to avoid selecting this distractor. These results confirm our original assumption, that the aqua-blue distractor was indeed more similar to the aqua target than the blue distractor, and rules out the possibility that the blue distractor in Experiment 1 captured most strongly because it was more similar to the target than the aqua-blue distractor.

These results are in line with previous findings showing that attention can be tuned to a specific feature value when relational search is impossible. Deviating from earlier findings, the present study did not show the strongest capture by the target-similar distractor (e.g., Becker et al., 2014; Harris et al., 2013), but by a distractor that was shifted away from the nontarget color. A possible reason for this discrepancy may be differences in the methods used to encourage feature-specific tuning: In previous studies, feature-specific tuning to the exact color value was encouraged by training observers to select an orange target among an equal number of red-orange and yellow-orange nontargets (Becker et al., 2014), or by showing the target randomly among all-red or all-yellow nontargets (Harris et al., 2013). Because the target was equally likely to be surrounded by red(dish) or yellow(ish) nontargets, the conditions were symmetrical and there was no incentive to shift attention to a different feature value. By contrast, in Experiment 2, the aqua target was surrounded by five greenish nontargets and a single blueish (aqua-blue or blue) distractor. These asymmetrical conditions may have prompted tuning attention to color that was shifted away from the nontargets, to achieve better discriminability of the target against the nontarget colors (because erroneously selecting the nontargets would have produced larger costs than selecting the distractor). Although this explanation drawn on the principles and mechanisms of an optimal tuning account, the present results indicate a much larger shift and wider tuning function than reported in previous optimal tuning studies (e.g., Navalpakkam & Itti, 2007; Scolari & Serences, 2009, 2010; Yu & Geng, 2019). Possible reasons for this discrepancy will be discussed in the following section.

## General discussion

The present study yielded several important results: First, in Experiment 1 we showed that a relatively matching stimulus captures most even if it is very dissimilar from the target, and a large distance away from the target. This confirms that attention is tuned to the relative feature of the target in a standard visual search task for a salient color target.

Previous studies had already shown that attention is tuned to the relative feature of the target, and not an optimal feature value or the target feature value in the spatial cueing paradigm (e.g., Becker et al., 2010, 2013, 2017; Becker, Atalla & Folk, in press; Harris et al., 2013; Schoenhammer et al., 2016). Moreover, previous visual search studies demonstrated that a relatively matching distractor captures attention and the gaze more strongly than a target-similar distractor, both when the colors vary along the red-yellow dimension (e.g., Becker, 2010; Becker et al., 2014) and when they vary along the green-blue dimension (Martin & Becker, 2018). However, the visual search results could be alternatively explained by an optimal tuning account or a combined similarity-saliency account.

In the present visual search study, we rendered the relatively matching distractor far more dissimilar to the target than in previous studies, so that they were outside the region of optimal tuning and/or tuning to target-similar colors. Despite this, we still observed the strongest capture effect by the relatively matching, blue distractor. This finding also cannot be attributed to bottom-up saliency effects: To gauge possible contributions of bottom-up saliency to capture, as we also included a highly salient, red distractor, and compared selection rates with a nonsalient, gray distractor. In the bluer target condition of Experiment 1, the red distractor did not show higher selection rates than the nonsalient gray distractor, effectively ruling out that capture was (strongly) determined by bottom-up saliency.

With this, strong capture by the relatively matching, blue distractor has to be attributed to how attention was top-down tuned to the target. Thus, the results provide strong evidence for the relational account, that attention is tuned to the relative feature of the target (e.g., bluer or bluest item), and that irrelevant items can capture attention independently of target similarity (Becker, 2010; Becker et al., 2010, 2013).

A combined similarity/saliency account or optimal tuning account cannot explain the findings of Experiment 1, as they centrally propose that attention is tuned to the exact target feature value (aqua; e.g., Duncan & Humphreys, 1989; Martinez-Trujillo & Treue, 2004), or a feature value that is slightly shifted away from the nontargets (to an optimal position; e.g., Navaloakkam & Itti, 2007; Scolari & Serences, 2009, 2010). Specifically, an optimal tuning account centrally proposes a spatially limited shift of attention away from the nontargets that can be maximally 0.075 x/y-units away from the target in CIE space (based on the data of Navalpakkam & Itti, 2007). The relatively matching, blue distractor in Experiment 1 was, however, more than twice this possible maximum distance away from the target (0.190 x/y-units in CIE space), ruling out an optimal tuning account.

Experiment 2 tested an alternative explanation of the optimal tuning account and a combined similarity/saliency account – that is that the relatively matching, blue distractor was perhaps more similar to the target than other distractors that were nominally more similar to the target (based on distance measures in CIE color space). Contrary to this possibility, the results of Experiment 2 showed that the perceptual similarity between the colors, as prescribed by CIE space, matched the capture rates of the different colors when observers were encouraged to tune attention to the target color. These results demonstrate the relatively matching, blue distractor was indeed perceptually dissimilar to the target, and show that capture by this distractor cannot be explained by optimal tuning or a combined similarity/saliency account. With this, the results of Experiment 1 provide the first evidence that visual selection in visual search, as measured by gaze capture, is also determined by the top-down tuning to the relative color of the target, not an optimal color or the target color itself.

In sum, the results of Experiment 1 are in line with previous spatial cueing studies that supported the relational account and ruled out an optimal tuning and a combined similarity-saliency account (e.g., Becker et al., 2013). With this, Experiment 1 confirms that covert visual attention (e.g., in spatial cueing; Schönhammer et al., 2016) and eye movements (e.g., Becker et al., 2014) operate on the same relational principle, thus confirming a close relationship between attention and eye movements (e.g., Deubel & Schneider, 1996).

However, this conclusion about Experiment 1 still leaves two questions open; first, how we should explain the results of Experiment 2; and especially, whether the results of Experiment 2 support optimal tuning? Second, how do we explain the results of previous optimal tuning studies suggesting a spatially limited shift of attention to an optimal color? These questions will be addressed in turn below.

### Optimal tuning

In the critical distractor conditions of Experiment 2, we encouraged tuning attention to the target feature value (aqua) by blocking the distractor conditions and repeatedly presenting a critical distractor (i.e., blue or aqua-blue distractor). The results showed stronger capture by an intermediate, aqua-blue distractor than by a target-similar (aqua) and blue distractor. Thus, in Experiment 2, attention was probably tuned closely to the aqua-blue color, that is, shifted 0.119 x/y-units away from the target in CIE space in the opposite direction from the nontargets. According to the optimal tuning account, tuning to a color that is shifted away from the nontargets can be more optimal because it facilitates discriminating the target from the nontargets (e.g., Geng, DiQuattro & Helm, 2017; Navalpakkam & Itti, 2007; Scolari & Serences, 2009; Yu & Geng, 2019), and this explanation can very well account for the finding that attention was shifted away from the nontargets to a more aqua-blue color.

However, the finding that the blue distractor also still attracted the gaze quite strongly (to the same extent as the target-similar, aqua distractor) suggests that the tuning functions are wider than originally proposed on the optimal tuning account (e.g., Navalpakkam & Itti, 2007). Moreover, tuning attention to aqua-blue means that attention was shifted ∼0.119 x/y units away from the target in CIE space, substantially more than 0.03 x/y-units away from the target, which was the magnitude of the attentional shift estimated by Navalpakkam and Itti (2007) on the basis of their study.

Therefore, an optimal tuning account would need to be modified to allow much larger shifts in attentional tuning and wider tuning functions to account for the findings of Experiment 2. Although it may be possible to modify the optimal tuning account along these lines, it is perhaps not advisable.

Of note, Navalpakkam and Itti (2007) studied optimal tuning in a perceptual decision task in which observers had to report the target position, and found that a probe with a slightly shifted nontarget color was confused with the target (see also Scolari & Serences, 2009; Yu & Geng, 2019). Errors in perceptual decisions that involve mistaking another item for the target are plausibly limited to target-similar items that are highly confusable with the target. Larger shifts in tuning are implausible because observers are unlikely to regularly confuse a highly dissimilar stimulus with the target.

This points to an important difference between the perceptual decision task and the present study, where we centrally measured visual selection by assessing eye movements to an irrelevant distractor. Erroneously selecting a distractor in visual search does not imply that it is perceptually confusable with the target or that it could be mistaken for the target after allocating attention to it. In fact, previous eye movement studies often found higher selection rates for relatively matching target-dissimilar distractors, but shorter dwell times, reflecting that target-dissimilar distractors can be rejected earlier after they have been selected (e.g., Becker et al, 2014; Martin & Becker, 2018). These results indicate a dissociation in preattentive and attentional/perceptual processes, whereby early, preattentive processes driving visual selection are largely independent of target similarity, whereas later, perceptual processes depend on target similarity. The perceptual decision task used in optimal tuning studies plausibly taps into these later, perceptual processes, which show relatively narrow tuning, and a moderate shift to target-similar colors that are potentially confusable with the target.

In sum, it seems that perceptual decision-making operates on a spatially very limited, narrow tuning function, and produces only a small shift to target-similar features (e.g., Navalpakkam & Itti, 2007; Scolari & Serences, 2009, 2010), whereas early processes of visual selection (e.g., attention, eye movements) are based on much wider tuning functions and potentially larger shifts in feature-specific search (i.e., when relational search is not feasible). Thus, although we can use the optimization principles and general mechanisms of optimal tuning to explain feature-specific tuning of attention, the operating parameters of early visual attention probably differ from those of perceptual decision-making, as identified in Navalpakkam & Itti (2007). In line with this conclusion, we also found that early visual selection was biased to the relative target feature in Experiment 1, in line with earlier studies reporting a preference for relational search (if this allows selecting the target on the majority of trials; Becker et al., 2013, 2014). Of note, relational search is probably not an optimal search strategy because it renders the searcher vulnerable to a wide range of different distractors (cf. Becker, 2010, Becker et al., 2014).

The differences between tuning of attention and perceptual decision making are probably rooted in the different affordances of the two visual functions: At the stage of perceptual decision making, it is important to correctly identify objects to allow successful interactions with objects. This purpose is perhaps best served by a narrow tuning function and only small deviations (shifts) away from the veridical target feature value (to optimize selection). At an early stage of visual selection, it is, however, more crucial to select the target or to avoid missing the target, even if this comes at the cost of selecting irrelevant distractors. This is a purpose perhaps best served by a relational search strategy or a wide, feature-specific tuning function that operates largely independent of target similarity. Tuning attention to the relative features of an object (e.g., larger, redder, darker) may be more successful than anchoring it to the exact feature value, because feature values in the hue, brightness, size and orientation dimensions vary dramatically with changes in the environment (e.g., changes in lighting, shading, perspective, distance).

These latter conclusions should still be regarded as tentative, however. As the present study probably did not encourage very narrow tuning to the target feature value, more research is necessary to characterize the width and position of tuning functions in visual search tasks. It is also possible that the width of the feature-specific tuning function is not fixed across different stimuli in a visual search task, but that it varies considerably, possibly depending on the feature value distribution of the irrelevant items (e.g., Itti & Koch, 2000; Martinez-Trujillo & Treue, 2004; Navalpakkam & Itti, 2007; Wolfe, 1994).

Although this question would require further research, the present study demonstrated that attention is tuned to the relative target feature in visual search when observers have to make eye movements. Moreover, Experiment 2 showed that minimal changes to the paradigm (i.e., blocking distractors) can change the shape of the tuning function and yield a different, feature-specific search strategy.

## Footnotes

1 It would also be interesting to inspect the saccade latencies for distractor fixations to examine possible differences in the time-course of distractor selection (e.g., Becker & Lewis, 2015; Becker, Lewis, et al., 2017), or the distractor dwell times (e.g., Martin & Becker, in press), to gauge how long it takes to identify each distractor as a nontarget item. However, these analyses could not be conducted in the present study because most distractors attracted gaze only weakly, leading to missing values and/or too few occurrences for these analyses.
